# Chemiresistive Effect in Ti_0.2_V_1.8_C MXene/Metal Oxide Hetero-Structured Composites

**DOI:** 10.3390/s26020496

**Published:** 2026-01-12

**Authors:** Ilia A. Plugin, Nikolay P. Simonenko, Elizaveta P. Simonenko, Tatiana L. Simonenko, Alexey S. Varezhnikov, Maksim A. Solomatin, Victor V. Sysoev, Nikolay T. Kuznetsov

**Affiliations:** 1 Department of Physics, Yuri Gagarin State Technical University of Saratov, 77 Polytechnicheskaya Str., 410054 Saratov, Russia; ilyaplygin@mail.ru (I.A.P.); alexspb88@mail.ru (A.S.V.); solomatin1994@gmail.com (M.A.S.); 2 Kurnakov Institute of General and Inorganic Chemistry of the Russian Academy of Sciences, 31 Leninsky pr., 119991 Moscow, Russia; ep_simonenko@mail.ru (E.P.S.); egorova.offver@gmail.com (T.L.S.); ntkuz@igic.ras.ru (N.T.K.); 3 Saratov Branch of Kotelnikov Institute of Radioengineering and Electronics of RAS, Zelenaya St. 38, 410019 Saratov, Russia

**Keywords:** MXene, chemiresistor, SnO_2_, Co_3_O_4_, V_2_O_5_, metal oxide, nanocomposite, synthesis, gas sensor, multisensor array

## Abstract

**Highlights:**

**What are the main findings?**

- Heterostructures of Ti_0.2_V_1.8_C MXenes with metal oxides have been synthesized.
- Ti_0.2_V_1.8_C MXene/metal oxide composites have been tested as chemiresistive elements.

**What are the implications of the main findings?**

- The synthesis protocol for Ti_0.2_V_1.8_C MXene/metal oxide composites could be further scaled when developing sensor prototype units.
- The Ti_0.2_V_1.8_C MXene/metal oxide composites are shown as promising, versatile materials for designing multisensor arrays.

**Abstract:**

Two-dimensional carbide crystals (MXenes) are emerging as a promising platform for the development of novel gas sensors, offering advantages in energy efficiency and tunable analyte selectivity. One of the most effective strategies to enhance and tailor their functional performance involves forming hetero-structured composites with metal oxides. In this work, we explore a chemiresistive effect in double-metal MXene of Ti_0.2_V_1.8_C and its composites with 2 mol. % SnO_2_ and Co_3_O_4_ nanocrystalline oxides toward feasibility tests with alcohol and ammonia vapor probes. The materials were characterized by simultaneous thermal analysis, X-ray diffraction analysis, Raman spectroscopy, and scanning/transmission electron microscopy. Gas-sensing experiments were carried out on composite layers deposited on multi-electrode substrates to be exposed to the test gases, 200–2000 ppm concentrations, at an operating temperature of 370 °C. The developed sensor array demonstrated clear analyte discrimination. The distinct sensor responses enabled a selective identification of vapors through linear discriminant analysis, demonstrating the further potential of MXene-based materials for integrated electronic nose applications.

## 1. Introduction

The growing societal need to have on-site knowledge of environmental conditions, essential for human well-being, is driving intensive study efforts toward the development of portable electronic devices that are capable of sensing and processing such information [1]. One of the major challenges in this field is providing users with real-time insights into the surrounding gas compositions and concentrations of specific target analytes [2]. To address the task, gas sensors have become critical components, prompting extensive research and development activities in numerous laboratories [3,4,5].

Among various sensing technologies, chemiresistive gas sensors, primarily introduced in the 1950s, are recognized for their simplicity and efficiency across a wide range of applications [6,7]. Presently, commercially available chemiresistors typically utilize polycrystalline metal oxides as the active sensing material [8,9]. However, recent advances in materials science, especially through the emergence of nanotechnology, have enabled the design of materials with one-, two-, and three-dimensional ordered architectures [10,11,12]. These nano-engineered materials offer significant potential for sensor applications. Notably, structures with at least one nanoscale dimension, comparable to the Debye length, ensure efficient interaction with the surrounding atmosphere and enable effective transduction of surface charge variations into measurable changes in electrical resistance [13,14].

Within a family of low-dimensional nanostructures, (quasi)-two-dimensional (2D) sheets are especially promising for integration into microelectronic platforms, which are predominantly planar and suitable for mass-scale production [15]. The first discovery of graphene layers sparked considerable interest, demonstrating chemiresistive behavior with the remarkable sensitivity to detect even single-molecule adsorption events under vacuum conditions [16]. Inspired by graphene, a new class of 2D materials, known as MXenes, carbide or nitride planar crystals, was introduced in 2011 [17]. These materials feature a layered structure consisting of metal and carbon/nitrogen sheets, and their inherent structural versatility and surface functionalization capabilities make them highly attractive for various applications, particularly in energy storage, catalysis [18,19,20], and gas sensing [21,22].

To further tailor the functional properties of MXenes in line with established trends in semiconductor technologies, these structures are doped with other materials to manufacture complex composites with enhanced performance characteristics [23]. As highlighted in recent reviews [24,25], MXene-based composites often outperform their pristine counterparts. Notably, the secondary phase in these composites typically consists of wide-bandgap oxides, which may form either through the natural oxidation of MXenes during thermal treatment or by deliberate addition during synthesis [26]. Tin dioxide (SnO_2_), an n-type semiconductor widely used in commercial gas sensors, is among the most common additives to such heterostructures [27,28,29]. However, p-type oxides such as cobalt ones (Co_3_O_4_) have also emerged as promising candidates for further exploration [30]. Herein, we consider even a more complex structure derived by employing a double-metal MXene of Ti_0.2_V_1.8_C [31] to test its composite via combining with these distinctive oxide semiconductors, SnO_2_ and Co_3_O_4_, as a functional layer for gas sensors of chemiresistive type and on-chip multisensor array. The tin and cobalt oxide content of 2 mol.% was chosen based on the formation of corresponding composites, where these compounds would develop a thin layer of nanoparticles on the surface of the base material while retaining their electrical conductivity.

## 2. Materials and Methods

### 2.1. Synthesis and Characterization of Materials Under Study

For the synthesis of the initial reagents and composite of Ti_0.2_V_1.8_C MXene samples modified with 2 mol.% SnO_2_ and Co_3_O_4_, titanium powders (>99%, LLC Snabtekhmet, Moscow, Russia), vanadium (99.9%, 0.5–100 μm, LLC RusKhim, Russia), aluminum (≥98%, LLC RusKhim, Moscow, Russia), graphite (>99.99%, LLC Osobo chistye veshchestva, Russia), KBr (>99%, LLC RusKhim, Russia), hydrofluoric acid (50%, chemically pure, Micropur ULSI, Switzerland), hydrochloric acid HCl (>99%, LLC RusKhim, Russia), 1-butanol (>98%, LLC TD Khimmed, Russia), cobalt(II) nitrate hexahydrate Co(NO_3_)_2_·6H_2_O (>99%, LLC TD Khimmed, Russia), and urea CH_4_N_2_O (>99%, LLC TD Khimmed, Russia) were used.

The synthesis of Ti_0.2_V_1.8_C MXene follows the protocol that was described earlier [31]. In brief, the corresponding Ti_0.2_V_1.8_AlC MAX phase was obtained as the starting reagent by synthesis in a salt melt, *n*(Ti):*n*(V):*n*(Al):*n*(C) = 0.2:1.8:1.2:0.8, >99% purity at 1100 °C. The Ti_0.2_V_1.8_C sample was obtained by selective etching of aluminum from the Ti_0.2_V_1.8_AlC MAX phase using a mixture of concentrated hydrofluoric and hydrochloric acids in a 3:2 volumetric ratio for 90 h at a temperature of 90 ± 3 °C. The product was then washed with distilled water to a pH of 5–6, separated by centrifugation at 3500 rpm, and vacuum-dried at 80 °C.

The Ti_0.2_V_1.8_C sample was modified with nano-dispersed SnO_2_ according to the previously described algorithm [32,33] during the hydrothermal synthesis of tin dioxide in the presence of dispersed multilayered MXene. In general, hydrothermal conditions facilitate the formation of anisotropic nanostructures and develop the surface of the material, which is especially important when designing resistive-type gas sensors. By meeting the required parameters of the applied procedures, we could ensure that the electrical characteristics of the composite materials were in good agreement with the literature [34]. Specifically, a Ti_0.2_V_1.8_C dispersion in ethanol with a concentration of 10 g/L was introduced into the calculated volume of a SnCl_2_ solution diluted with hydrochloric acid, to which a 5% aqueous solution of NH_3_·H_2_O was then added until the pH reached 9. Hydrothermal synthesis was carried out in a 5 mL steel autoclave with a Teflon liner at a temperature of 120 °C for 1 h. The resulting solid product, Ti_0.2_V_1.8_C/SnO_2_, was separated by centrifugation, washed with distilled water to a pH of 5–6, and then washed once with ethanol.

The preparation of the Ti_0.2_V_1.8_C/Co_3_O_4_ composite is similar to that described for the decoration of Ti_2_C MXene, as reported elsewhere [35]. For this purpose, a Ti_0.2_V_1.8_C dispersion of 10 mg in 1 mL 1-butanol was introduced into the calculated volume of a 0.05 M solution of cobalt(II) nitrate and 0.25 M urea in ethanol. The resulting system was transferred to a 5 mL steel autoclave with a Teflon insert and hydrothermally treated at 160 °C for 1 h. The solid phase was separated by centrifugation and washed twice with ethyl alcohol.

The resulting composite powders of Ti_0.2_V_1.8_C/SnO_2_ and Ti_0.2_V_1.8_C/Co_3_O_4_ were dispersed in 1 mL of 1-butanol in an ultrasonic bath for 30 min. The derived dispersions were used as functional inks to obtain a receptor layer by microplotter printing. Sample drying was performed under vacuum conditions at a temperature of 80 °C.

The thermal behavior of the materials was studied using an SDT Q600 thermal analyzer (TA Instruments, New Castle, DE, USA) over a temperature range of 25–600 °C in an air flow of 250 mL/min with a heating rate of 10 degrees/min. In this case, during heating, the change in heat flow (differential scanning calorimetry analysis, DSC) and the mass of the analyzed sample (thermogravimetric analysis, TGA) were synchronously registered.

X-ray diffraction (XRD) analysis of the films, based on the materials under study, was performed with a D8-Advance diffractometer (Bruker, Billerica, MA, USA), CuKα radiation of 1.5418 Å wavelength, upon applying the Ni filter with the following parameters: E = 40 kV, I = 40 mA, signal accumulation time of 1.0 s/point, resolution of 0.06°, and 2θ range of 5–70°.

The microstructure was investigated using transmission electron microscopy (TEM; Jeol JEM-1011 microscope (Jeol Ltd., Tokyo, Japan) equipped with an Orius SC1000W digital camera) and scanning electron microscopy (SEM; Tescan Amber microscope, Tescan, s.r.o., Brno, Czech Republic). The chemical composition of the materials obtained after synthesis and heat treatment was analyzed using energy-dispersive X-ray spectroscopy (EDS, Ultim MAX EDS detector with an active crystal area of 100 mm^2^, Oxford Instruments Group, Oxfordshire, UK).

Raman spectra were recorded using a Confotec NR500 confocal Raman microscope (SOL Instruments, Augsburg, Germany) with a laser excitation wavelength of 532 nm, diffraction grating of 600 lines/mm, ×20 objective, and NA = 0.50.

### 2.2. Gas-Sensing Characterization of On-Chip Sensor Elements

To assess the chemiresistive performance of MXene/metal oxide heterostructures, suspensions of the active materials (5 wt.%) were printed onto Si/SiO_2_ substrates equipped with multiple co-planar strip Pt electrodes [36] of a 50 μm width and 1 μm thickness using a microplotter. The materials under study were deposited to ensure the presence of percolation tracks of the nanosheet particles in the layer between the electrodes. The fabricated structures included pure Ti_0.2_V_1.8_C as a reference and its composites containing 2 mol.% of either SnO_2_ or Co_3_O_4_. The chips, 9 × 10 × 0.38 mm^3^, also featured the integrated meander-type Pt heaters and thermoresistors.

**Figure 1:**
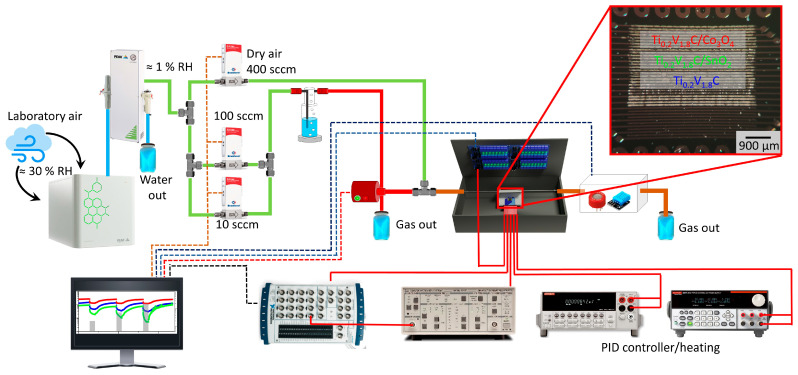
The experimental setup to measure the chemiresistive effect in MXene/metal oxide composite-based sensor chips. The dashed and solid red/blue lines indicate electrical connections between the units.

The assembled sensor chips were mounted within a sealed stainless steel chamber designed for dynamic gas-flow experiments. The scheme of the entire experimental setup is shown in Figure 1. Dried laboratory air, with a humidity level below 200 ppm, was supplied at a rate of 400 sccm using a dry-air generator (Peak Purge PG14L, Peak Scientific Instruments, Glasgow, UK) and an air compressor (Precision Compressed Air 230B, Peak Scientific Instruments, Glasgow, UK). This flow was split into two paths: one directed through bubblers containing aqueous solutions of the target analytes—methanol, ethanol, butanol, and 20% aqueous ammonia —and the other serving as a dilution reference. Analyte concentrations of the volatile organic compounds (VOCs) were precisely regulated at a 200–2000 ppm range using mass-flow controllers (Bronkhorst, Ruurlo, The Netherlands). Gas composition was validated by an auxiliary commercial sensor (MQ-3, Shenzhen, China) and a hygrometer (DHT AM2302, Shenzhen, China) at the gas exit to confirm the presence/intensity of VOC and humidity levels, respectively.

Electrical measurements were performed under a constant 5 V of d.c. bias using a custom-built acquisition system. I–V characteristics were recorded in the range of [−5, +5] V in 0.5 V increments. On-chip sensor elements were sequentially addressed using an analog multiplexer (SRD-05VDC-SL-C, Songle Relay^®^, Yuyao City, China), and current responses were amplified and digitized via a preamplifier (SRS570, Stanford Research System, Sunnyvale, CA, USA) wired to the input/output multi-pin module (NI-DAQ USB-6259, National Instruments, Austin, TX, USA) to measure the resistance of the on-chip material between each pair of electrodes. The temperature was maintained up to 370 °C using Keithley instruments (2230-30-1 current source, 2000 multimeter, Solon, OH, USA) with 2.4 W of maximum power consumption. The entire equipment was controlled by a personal computer under LabView^@^ software (LabView Full Development System with Application builder, ver. 2020 SP1, National Instruments Co., Austin, TX, USA).

## 3. Results and Discussion

### 3.1. Results of Physical Characterization of the Materials

**Figure 2:**
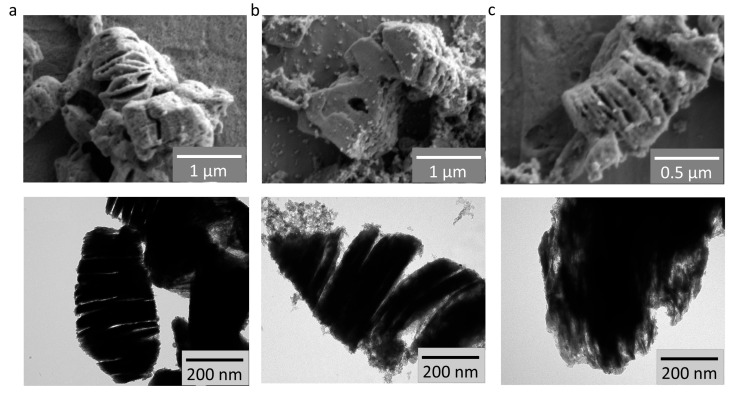
The scanning and transmission of electronic images taken from three samples of Ti_0.2_V_1.8_C (**a**), Ti_0.2_V_1.8_C/SnO_2_ (**b**), and Ti_0.2_V_1.8_C/Co_3_O_4_ composites (**c**).

For further evaluation, identical films based on the materials under study were formed on glass substrates, which were subsequently heat-treated at 370 °C for 2 h under an air environment. SEM and TEM images for all samples confirmed the layered structure inherited from Ti_0.2_V_1.8_C MXene (Figure 2). Additionally, the formation of smaller particles related to the corresponding dopants in Ti_0.2_V_1.8_C/SnO_2_ and Ti_0.2_V_1.8_C/Co_3_O_4_ composites was observed on the surface of quasi-two-dimensional structures.

**Figure 3:**
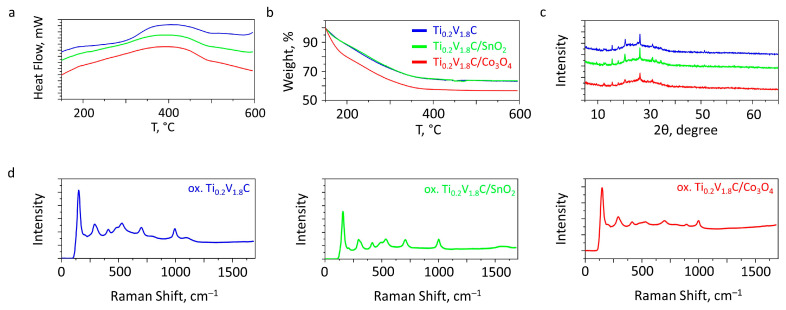
The physical characterization of three layers of Ti_0.2_V_1.8_C, Ti_0.2_V_1.8_C/SnO_2_, and Ti_0.2_V_1.8_C/Co_3_O_4_: (**a**) DSC, (**b**) TGA, (**c**) XRD analysis, and (**d**) Raman spectroscopy.

Synchronous thermal analysis data indicate that the oxidation process, characterized by an energy release (Figure 3a) and mass loss (Figure 3b), is nearly complete when the materials are heated in air up to 370 °C. The results of the XRD analysis (Figure 3c) and Raman spectroscopy (Figure 3d) of the materials after heat treatment at 370 °C indicate the formation of vanadium (V) oxide as the major component, in good agreement with the thermal analysis data. Thus, a set of reflections located near 15.6°, 20.6°, 26.3°, and 31.2°, corresponding to the {200}, {001}, {110}, and {301} crystallographic planes, respectively, can be observed in the X-ray diffractograms. This diffraction pattern is characteristic of the presence of orthorhombic-structured V_2_O_5_ [37]. A low-intensity signal near 12.5° can be associated with the presence of a minor Ti_0.2_V_1.8_AlC MAX phase impurity [38]. In all cases, the Raman spectra exhibit modes at around 149, 202, 291, 414, 489, 529, 698, and 1001 cm^−1^, which are related to the bending and stretching vibration of the vanadium (V) oxide bonds. The presence of an additional signal at around 891 cm^−1^ may be due to the existence of the amorphous V_2_O_5_ phase in the material composition [39].

### 3.2. Results of Gas-Sensing Characterization of On-Chip Sensor Elements

The initial measurements at room temperature revealed a negligible chemiresistive response of all three MXene composite structures to VOC analytes, prompting heating up to 370 °C, which is known to properly activate metal oxides for observing a chemiresistive effect [40].

Under these conditions, partial oxidation of Ti_0.2_V_1.8_C into Ti_0.2_V_1.8_C/V_2_O_5_ was confirmed according to the results given in Section 3.1 in accordance with previous findings [41]. The I–V curves for all the materials displayed Ohmic behavior, with a power law $I\sim U^{b}$, where exponents *b* were equal to 1.04 (ox. Ti_0.2_V_1.8_C), 1.07 (Ti_0.2_V_1.8_C/Co_3_O_4_), and 1.20 (Ti_0.2_V_1.8_C /SnO_2_), indicating minimal potential barriers at the electrode interfaces.

**Figure 4:**
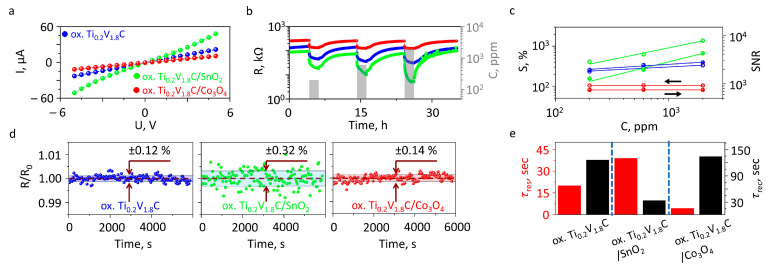
Electrical/chemiresistive characterization of three layers of oxidized Ti_0.2_V_1.8_C, Ti_0.2_V_1.8_C/SnO_2_, and Ti_0.2_V_1.8_C/Co_3_O_4_ as a part of multi-electrode chip: (**a**) I–V curves; (**b**) R(t) transients recorded upon exposure to ethanol vapors, of various concentrations; (**c**) chemiresistive response and signal-to-noise ratio (SNR) *versus* ethanol concentration; (**d**) resistive noise; (**e**) characteristic response/recovery times.

During gas exposure tests, resistance changes were monitored over a 2 h exposure period followed by an 8 h air purging of the tubes/camera. The resistance of all the sensor elements in the on-chip array varied in the range of hundreds of kOhm to suit ordinary electronic reading schemes in accordance with the I–V measurements. The typical R(t) transients recorded upon interaction with ethanol vapors are drawn in Figure 4b.

As one can see, the low-noise resistance reproducibly changes following the adsorption of analyte molecules. It is worth noting here that the alcohol VOCs forced the resistance of all three hetero materials to go down, while ammonia resulted in increasing resistance. The observed differences are attributed to the complex nature of the composites under study, where all the elements synergistically contribute to the integral chemiresistive effect [42,43,44]. The higher concentration of the analytes leads to a greater chemiresistive response that is calculated as a relative change of resistance:

(1) $$S=\left(\frac{G_{gas}}{G_{air}}-1\right)\cdot 100\%,$$

where *G_gas_* and *G_air_* are the sensor conductance recorded upon exposure to gas analytes and background air, respectively. The derived empirical dependencies of *S versus* analyte concentration recorded for all three composite samples followed a power law, $S\sim C^{n}$, to be consistent with the Freundlich isotherm.

The coefficients of *n*, characterizing the interaction between the hetero materials under study and analytes, are collected and shown in Table 1. As one can see, these values vary in a wide range, demonstrating significant variations among the MXene composites. These variations clearly arise from the formation of heterojunctions between the MXene structure and the oxides, which can be primarily attributed to the differences in the work functions of the interfaced materials [45,46,47].

The nature of the junctions, potential barrier magnitude, and induced charge transfer that occurred in these contacts require further thorough fundamental study. However, from a practice viewpoint, the observed variations are crucial when considering the layers to constitute a multisensor array for selective gas recognition, as we discuss further.

Another sensor characteristic, necessary for any practice application, is a signal-to-noise ratio (SNR), which is calculated as

(2) SNR=S/noise,

(3) $$noise=(\frac{\sigma }{R_{med}})\cdot 100\%,$$

where *S* is a chemiresistive response, σ is the standard deviation of resistance *R*, and *R_med_* is the median resistance value taken in a stabilized *R*(*t*) curve.

We have estimated the resistive noise in the sensor elements of the three MXene/metal oxide composites, primarily in the dry-air conditions. These data are given in Figure 4d. The lowest noise is observed in oxidized Ti_0.2_V_1.8_C MXene, where the resistance variation is ca. 0.12% relative to a median resistance, and the addition of cobalt oxide, 2 mol. %, into this MXene resulted in a slight enhancing of the noise to reach ca. 0.14%. In contrast, tin dioxide introduced a more significant increase in the noise—up to values of ca. 0.32%—which possibly reflects larger barriers appearing in MXene/metal oxide heterojunctions due to the differences in work functions of these materials. Interestingly, these observations correlate with the I–V behavior of these materials (Figure 4a), where the most significant curve bending is for the MXene/SnO_2_ composite.

Accounting for the noise data, we extracted SNR values, which were plotted in Figure 4c. In the measured concentration range of analytes, 200–2000 ppm, SNR data characterizing all the test gases significantly exceeded 3 un., which is considered to be minimum for the practical use of sensors. In addition, the graph shows that a lower noise yields the oxidized MXene of Ti_0.2_V_1.8_C to have a higher SNR, although its chemiresistive response is lower when compared to the Ti_0.2_V_1.8_C/SnO_2_ composite.

Furthermore, we have analyzed the response and recovery times as

(4) $$\frac{G(t)}{G_{0}}=1-exp\left(-\frac{t}{t_{res}}\right)$$

(5) $$\frac{G(t)}{G_{0}}=exp\left(-\frac{t}{t_{rec}}\right),$$

where *G*_0_ and *G*(*t*) are the conductivity of the sensor element in the background atmosphere and the presence of analyte vapors, respectively, and *t_res_* and *t_rec_* are the response and recovery times.

These derived values for the three samples under study are plotted in Figure 4e to lie in a minute range. We may note that the sample of Ti_0.2_V_1.8_C/SnO_2_ is characterized by an increased response time and reduced recovery time when compared to the oxidized Ti_0.2_V_1.8_C MXene structures. At the same time, the Ti_0.2_V_1.8_C/Co_3_O_4_ sample has the lowest response time, of ca. 3.8 s, but the highest recovery time. These features require a further thorough investigation to possibly derive them from variations in the adsorption energy of various analytes over the hetero-structured composites under study.

**Figure 5:**
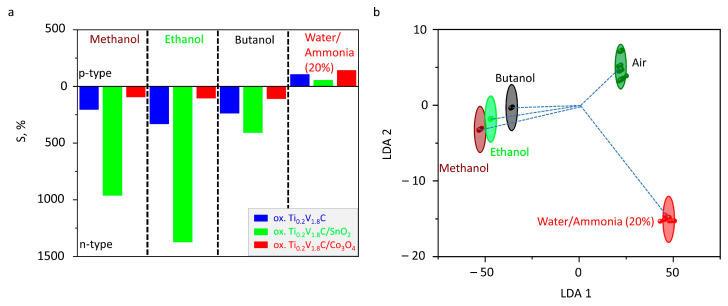
Gas selectivity performance of three layers of oxidized Ti_0.2_V_1.8_C, Ti_0.2_V_1.8_C/SnO_2_, and Ti_0.2_V_1.8_C/Co_3_O_4_ to alcohols and NH_3_ (2000 ppm concentration) as a part of a multi-electrode chip: (**a**) chemiresistive response and (**b**) the results of linear discriminant analysis; points are empirical vector responses, and ellipses frame the scatter of in-class data under a Gaussian distribution with a 0.9 confidence level.

Comparing the chemiresistive responses of these three composite structures toward test analytes (Figure 5a), we should note that oxide additives significantly affect the selectivity. An addition of SnO_2_ enhances the composite response to alcohols but surpasses it to ammonia vapors, whereas Co_3_O_4_ promotes an opposite behavior, advancing a response to ammonia and lowering it to alcohols. Accounting for observed noise data, the latter one indicates an improvement in sensitivity and a limit of detection in the Ti_0.2_V_1.8_C/Co_3_O_4_ sample. These complementary behaviors support utilizing the sensor array to be composed of layers derived from various composites to broaden detection capabilities.

Altogether, the variations in selectivity observed throughout various composite layers in the on-chip sensor elements yield one option to employ it in the framework of multisensor array analysis according to the electronic nose concept [48]. With this purpose, we have applied linear discriminant analysis (LDA) to process the on-chip array response to test analytes. This technique transfers the original multidimensional data to the (*N*-*1*)-dimensional space, where *N* is the number of analytes to recognize [49].

The analyte distinction features extracted by the method are placed along axes called LDA components. Figure 5b shows the 2D projection of the entire 4D LDA space built following processing of the MXene/metal oxide composite-based on-chip multisensor array. As one can see, the vector responses to various analytes (2000 ppm concentration) are clustered in distinguished spots of the LDA space so as to be distanced from each other and the center of the coordinate system. We have circled these areas around gravity centers under a Gaussian distribution of in-class data with a 0.9 confidence level [50]. It allows one to selectively identify the test gas by eye or via conventional machine learning algorithms.

## 4. Conclusions

This work demonstrates the potential of Ti_0.2_V_1.8_C-based MXene composites with SnO_2_ and Co_3_O_4_ for the selective detection of VOCs and ammonia, as exemplary analytes, via chemiresistive sensing. An elevated temperature operation of about 370 °C enables the activation of oxide components and oxidation of MXene to V_2_O_5_-rich structures, enhancing the gas response. While SnO_2_ increases sensitivity to alcohols, Co_3_O_4_ favors ammonia detection; both modifications impact the noise characteristics and kinetics of sensor response. Integration of these materials into a single chip enables analyte discrimination using linear discriminant analysis, confirming their applicability in electronic nose systems for environmental monitoring, industrial safety, or medical diagnostics. However, further research is required (i) to advance protocols for depositing composite materials over commercially available chip-based platforms in frames of state-of-art mass-scaled microelectronic technologies, (ii) clarify the number of analyte gases that are capable of detection with the help of materials beyond ones that are probed in this feasibility study, and (iii) test the long-term stability of the material’s response under normal and harsh air conditions in order to ascertain their constraints when compared to other known sensor-based materials.

## Figures and Tables

**Table 1:** The power law index, n, extracted from the dependence the dependence of the chemiresistive response of three composite materials *versus* an analyte concentration in the 200–2000 ppm range.

Analyte	MXene/Metal Oxide Composite		
	Oxidized Ti_0.2_V_1.8_C	Ti_0.2_V_1.8_C/SnO_2_	Ti_0.2_V_1.8_C/Co_3_O_4_
Ammonia	0.43 ± 0.17	0.18 ± 0.24	0.81 ± 0.09
Methanol	0.07 ± 0.002	0.32 ± 0.08	0.02 ± 0.002
Ethanol	0.15 ± 0.03	0.58 ± 0.07	0.001 ± 0.005
Butanol	0.20 ± 0.02	0.13 ± 0.05	0.06 ± 0.01

## Data Availability

The raw data supporting the conclusions of this article will be made available by the authors upon request.
